# Validation of an Artificial Intelligence Model for Breast Cancer Molecular Subtyping Using Hematoxylin and Eosin-Stained Whole-Slide Images in a Population-Based Cohort

**DOI:** 10.3390/cancers17193234

**Published:** 2025-10-05

**Authors:** Umay Kiraz, Claudio Fernandez-Martin, Emma Rewcastle, Einar G. Gudlaugsson, Ivar Skaland, Valery Naranjo, Sandra Morales-Martinez, Emiel A. M. Janssen

**Affiliations:** 1 Department of Pathology, Stavanger University Hospital, 4011 Stavanger, Norway; emma.rewcastle@sus.no (E.R.); einar.gudbjorn.gudlaugsson@sus.no (E.G.G.); ivar.skaland@sus.no (I.S.); emilius.adrianus.maria.janssen@sus.no (E.A.M.J.); 2 Department of Chemistry, Bioscience and Environmental Engineering, University of Stavanger, 4021 Stavanger, Norway; 3 Instituto Universitario de Investigación en Tecnología Centrada en el Ser Humano, HUMAN-Tech, Universitat Politècnica de València, 46022 Valencia, Spain; clferma1@i3b.upv.es (C.F.-M.); vnaranjo@dcom.upv.es (V.N.); sanmomar@htech.upv.es (S.M.-M.); 4 Institute for Biomedicine and Glycomics, Griffith University, Southport, QLD 4215, Australia

**Keywords:** breast cancer, molecular subtype prediction, hematoxylin and eosin-stained, whole-slide images, computational pathology, deep learning

## Abstract

**Simple Summary:**

Breast cancer is a complex disease that can be classified into different biological subtypes. Correctly identifying these subtypes is essential in determining the most effective treatment for each patient. However, current methods such as gene expression testing and immunohistochemistry are either expensive, time-consuming, or not widely available in all healthcare settings. In this study, we explored whether a computer-based approach using artificial intelligence can accurately predict breast cancer subtypes by analyzing routine pathology slides stained with hematoxylin and eosin. This real-world validation study shows that this method can identify certain subtypes with promising accuracy, offering a faster and more accessible alternative to existing techniques. This research may help improve diagnostic processes, especially in hospitals with limited resources, and support more personalized treatment decisions for patients with breast cancer.

**Abstract:**

**Background/Objectives**: Breast cancer (BC) is the most commonly diagnosed cancer in women and the leading cause of cancer-related deaths globally. Molecular subtyping is crucial for prognosis and treatment planning, with immunohistochemistry (IHC) being the most commonly used method. However, IHC has limitations, including observer variability, a lack of standardization, and a lack of reproducibility. Gene expression profiling is considered the ground truth for molecular subtyping; unfortunately, this is expensive and inaccessible to many institutions. This study investigates the potential of an artificial intelligence (AI) model to predict BC molecular subtypes directly from hematoxylin and eosin (H&E)-stained whole-slide images (WSIs). **Methods**: A pretrained deep learning framework based on multiple-instance learning (MIL) was validated on the Stavanger Breast Cancer (SBC) dataset, consisting of 538 BC cases. Three classification tasks were assessed, including two-class [triple negative BC (TNBC) vs. non-TNBC], three-class (luminal vs. HER2-positive vs. TNBC), and four-class (luminal A vs. luminal B vs. HER2-positive vs. TNBC) groups. Performance metrics were used for the evaluation of the AI model. **Results**: The AI model demonstrated strong performance in distinguishing TNBC from non-TNBC (AUC = 0.823, accuracy = 0.833, F1-score = 0.824). However, performance declined with an increasing number of classes. **Conclusions**: The study highlights the potential of AI in BC molecular subtyping from H&E WSIs, offering an easily applicable and standardized method to IHC. Future improvements should focus on optimizing multi-class classification and validating AI-based methods against gene expression analyses for enhanced clinical applicability.

## 1. Introduction

According to the International Agency for Research on Cancer, breast cancer (BC) is the most diagnosed cancer and the leading cause of cancer death in women, and it was the second most common cancer overall in 2022 [1]. In today’s era, where the importance of personalized treatment is increasing, accurately determining the molecular subtype of BC is crucial for treatment planning and prognosis in clinical practice.

After Perou et al.’s groundbreaking publication in 2000 [2], which showed that BC could be classified into different subtypes based on gene expression, many other studies have been able to confirm their results. As of now, molecular subtyping based on gene expression patterns is considered the ground truth. Significant efforts have been made over the years to characterize and classify BC at the molecular level, aiming to refine classification and treatment strategies [3]. Cluster classifications are used for BC, such as hierarchical (intrinsic subtypes) and integrative (IntClust), therefore leading to different behaviors and prognoses [4]. Although several commercial tests based on gene expression [5] exist that can both classify patients into different subgroups and even predict treatment outcome and prognosis, IHC is still used in many countries due to the costs of the genomic tests. In some countries, the cost of IHC has to be paid by the patient itself; as such, IHC is not used at all in some parts of the world.

The surrogate IHC-based classification system categorizes BC into luminal A, luminal B, HER2-positive, and triple-negative BC (TNBC) according to estrogen receptor (ER), progesterone receptor (PR), proliferation (either Ki67 or mitosis counting), and human epidermal receptor growth factor 2 (HER2) expression.

These subtypes exhibit significant differences in incidence, treatment options, response to treatment, disease progression, and survival. Luminal A tumors exhibit high ER and PR expression and low levels of proliferation, resulting in excellent outcomes. In contrast, luminal B tumors are characterized by higher proliferation rates and lower PR and/or HER2 expression, leading to significantly poorer prognosis compared to luminal A tumors [6]. Luminal A cancers are typically treated with endocrine therapy, while luminal B cancers often are first treated with adjuvant chemotherapy followed by endocrine therapy based on tumor stage, menopausal status, and life expectancy [7]. HER2+ tumors have poor response to systemic chemotherapy and are treated with anti-HER2-targeted therapy such as trastuzumab [8]. Furthermore, TNBC is aggressive and has poorer outcomes compared to other BC subtypes. TNBC is not sensitive to hormone therapy or anti-HER2-targeted therapy; as such, chemotherapy is the main systemic treatment [9].

With the implementation of digital pathology in many Western countries and the rapid development of artificial intelligence (AI), important steps can be taken in pathology. These novel methods are required to develop an accurate and reproducible evaluation of predictive and prognostic biomarkers, avoid inter- and intra-observer variabilities, and refine personalized treatment, reducing overtreatment while not increasing undertreatment. As the abovementioned IHC and gene expression analyses still have challenges and limitations, we suggest the possibility of using an AI model to predict molecular subtyping from a hematoxylin and eosin (H&E)-stained slide. Such a model could, in principle, be more reproducible, cheaper, and faster than IHC or molecular tests. As such, in this study, we present a large population-based cohort of primary BC tumors from a single center in Norway, with their H&E-stained whole-slide images (WSIs) digitalized across three different centers. We validated an AI model with these data to test the hypothesis that the AI method is objective, sensitive, and specific when predicting molecular subtypes.

## 2. Materials and Methods

### 2.1. Dataset

**Figure 1:**
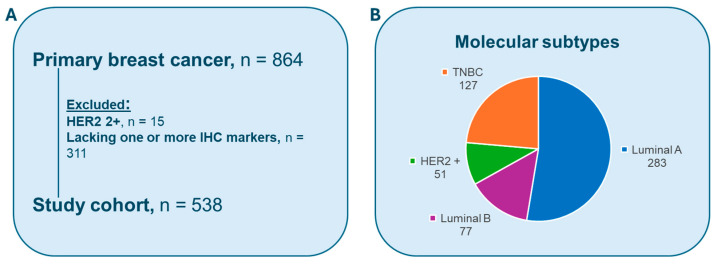
(**A**) Stavanger breast cancer dataset. (**B**) The patient distribution across the subtypes.

Stavanger Breast Cancer (SBC) Dataset: A total of 864 consecutive lymph node-negative BC patients diagnosed at Stavanger University Hospital in Norway (1978–2004) were originally considered. The following cases were excluded from the study: 15 cases of HER2 2+ by IHC, and 311 cases lacked one or more IHC markers for molecular subtyping in different classification tasks. As a result, 538 patients were included in the study (Figure 1A). Two expert pathologists (U.K. and E.G.) selected the most representative slide for each patient, and an initial quality check was performed manually. All H&E-stained slides were scanned at 40 magnifications using three different scanners, with 87 cases using Hamamatsu NanoZoomer S60 (.ndpi), 43 cases using Hamamatsu NanoZoomer 2.0HT (.ndpi) (Hamamatsu Photonics, Hamamatsu City, Japan), and 408 cases using Leica Aperio AT2 (.svs) (Leica Biosystems, Richmond, IL, USA).

### 2.2. Immunohistochemistry

ER, PR, HER2, and Ki-67 expression were determined by IHC in whole sections. ER, PR, HER2, and Ki67 IHC stains were performed and evaluated by a different observer for the previous studies using adequate quality controls according to the guidelines of the Norwegian Breast Cancer Group (NBCG) [10,11]. ER was scored as positive when nuclear staining was present in ≥1% and scored negative when it was <1% [3]. PR was scored as positive when nuclear staining was present in ≥10% and negative when it was <10% [10]. HER2 was scored between 0 and +3 depending on the membranous staining pattern and the intensity of the staining according to the WHO classification [3]. Cases with 0 and 1+ were evaluated as HER2-negative, while those with 3+ were evaluated as HER2-positive (HER2+). The cases with a 2+ expression of HER2 were excluded from the dataset, as additional information based on in situ hybridization was lacking. The Ki67 percentage was determined by the conventional Ki67 score, which has been previously described [12]. In short, the hotspot score is determined by examining the entire invasive region of the tumor section at low power to identify the most proliferative area. Once identified, both positive and negative nuclei are counted within a single field of view at high power (40X-objective). The Ki67 score is then calculated by dividing the number of positive Ki67 tumor nuclei by the total number of both positive and negative tumor nuclei, then multiplying by 100. The NBCG recommends counting a total of at least 500 tumor nuclei [10]. Due to the lack of consensus on the Ki67 threshold to distinguish low and high proliferation, we use the mitotic activity index (MAI) as a supplementary measure. According to the NBCG, Ki67 < 5% is classified as low proliferation, while Ki67 > 30% is considered high proliferation [10]. If Ki67 values are unavailable or fall between 5% and 30%, the MAI is used with a cutoff value of 10, with an MAI < 10 indicating low proliferation and an MAI ≥10 indicating high proliferation. The surrogate classification was made after the quantification of IHC (Table 1).

The patient distribution across the subtypes is presented in Figure 1B, with the majority classified as luminal A (n = 283, 53%), reflecting its higher prevalence in the population. Luminal B accounts for 77 patients (14%), HER2-positive cases total 51 (9%), and TNBC represents 127 cases (24%).

### 2.3. AI Model

**Figure 2:**
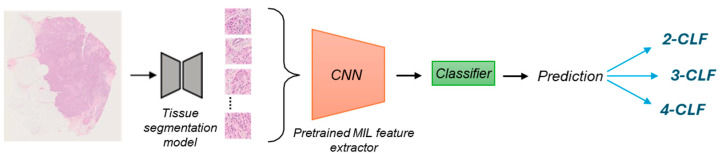
Method overview. Each whole slide image (WSI) was segmented to identify relevant tissue regions and divided into non-overlapping patches. These patches were processed using a pretrained convolutional neural network (CNN)-based multiple-instance learning (MIL) feature extractor, and the resulting features were subsequently passed to a classifier to generate task-specific predictions across different classification tasks (2-CLF, 3-CLF, or 4-CLF).

The AI model (Figure 2) employed in this study leverages a pretrained deep learning framework based on multiple-instance learning (MIL) principles to predict the molecular subtypes of BC directly from H&E-stained whole-slide images (WSIs). The MIL paradigm is particularly suited for this task, as it allows for weakly supervised learning using only slide-level labels without requiring detailed annotations at the patch or pixel level.

The model was trained on the publicly available Early Breast Cancer Core-Needle Biopsy Whole-Slide Image (BCNB) dataset [13]. This dataset includes 1058 core-needle biopsy WSIs with age, tumor size, tumor type, number of lymph node metastases, IHC results, and biopsy-level labels for molecular subtypes. It serves as the foundation for training the feature extractor. The backbone of the model is a convolutional neural network (CNN) pretrained under the MIL paradigm, utilizing the MIL attention mechanism [14]. This is a standard approach in the MIL literature that enhances the aggregation of patch-level features into slide-level representations. This attention mechanism ensures that the most discriminative patches contribute more significantly to the final prediction, improving the robustness of the feature extraction process.

To validate the generalizability of the model, we performed the validation using the SBC dataset. The SBC dataset provides an independent test set that reflects real-world variability in image acquisition and staining protocols, making it an ideal benchmark for evaluating the model’s clinical applicability.

#### 2.3.1. Feature Extraction on the SBC Dataset

Each WSI in the SBC dataset was subjected to several preprocessing steps before being used as input into the model. This included an automatic quality control assessment to address variations in staining and image acquisition across scanners as well as artifact exclusion. We normalize all slides to a consistent staining distribution so that cases from different scanners fall within the same color domain [15]. A tissue segmentation model [16] was used to filter out artifacts and non-informative patches, and only those with at least 60% tissue content were retained. This ensures that the analysis focuses on the biologically relevant regions of the WSIs. For each WSI, the tissue regions were divided into non-overlapping patches of size 512 × 512 pixels at 20× magnification.

The pretrained CNN-based MIL feature extractor was then used to compute deep feature vectors for informative patches. These feature vectors encapsulate the learned representations of tissue morphology and texture, which are critical for distinguishing between molecular subtypes. The extracted features were subsequently used to retrain the final classifier, adapting the model to the specific characteristics of the SBC dataset.

#### 2.3.2. Retraining the Final Classifier

The final classification layer of the pretrained model was fine-tuned using the SBC dataset to ensure optimal performance in this cohort. Both oversampling and undersampling strategies were applied, complemented by the application of a weighted categorical cross-entropy loss function during fine-tuning to address class imbalance within the dataset. The fine-tuned model was then evaluated across different molecular subtype classification tasks (CLF), including binary (two-class), three-class, and four-class settings, as summarized in Table 2.

#### 2.3.3. Performance Evaluation

The AI model’s performance was evaluated using standard metrics. The area under the curve (AUC) was used to assess the model’s ability to distinguish between different classes. Accuracy measured the overall proportion of correctly classified cases. To address potential class imbalance, the F1 score was employed, as it balances precision and recall. Additionally, precision and recall were analyzed individually to better understand the trade-off between false positives and false negatives.

Due to the absence of predefined training, validation, and test splits in the SBC dataset, a repeated k-fold cross-validation (CV) strategy was employed. This involved performing three repeats of 5-fold CV across all SBC cases. This approach ensures robust evaluation by systematically dividing the dataset into multiple training and validation folds and repeating this process with different random shuffles, thereby reducing the risk of overfitting and providing a more stable and reliable estimate of model performance.

For each fold, the pretrained feature extractor (backbone trained on the BCNB dataset) was used to extract deep feature vectors from the informative patches of the SBC WSIs. These features were then used to retrain the final classifier. A small hyperparameter search was conducted during this process to optimize the learning rate and weight decay for the classifier.

## 3. Results

The overall performance evaluation results of our MIL backbone pretrained on the BCNB dataset for classifying BC molecular subtypes across three different classification tasks are summarized in Table 3. These are the best overall mean results across the test sets of the 5-fold of the three repeats, choosing the models for each task with the best mean F1 score.

The model performed best in the 2-CLF task, with an AUC of 0.823, accuracy of 0.833, and a high F1 score of 0.824, suggesting strong differentiation between the two classes. However, as the number of classes increased, performance decreased. In the 3-CLF task, the model maintained a relatively high AUC (0.834), but accuracy dropped to 0.795 and the F1 score decreased to 0.770, indicating greater difficulty distinguishing among three molecular subtypes. The 4-CLF task demonstrated the most significant decline in performance, with the AUC dropping to 0.790, accuracy decreasing to 0.642, and the F1 score reaching only 0.601, highlighting the increased complexity in distinguishing among four BC subtypes. Precision and recall followed similar trends, with both metrics decreasing as the classification task became more complex, particularly in the 4-CLF setting, where precision and recall dropped to 0.587 and 0.642, respectively. While the model effectively classifies distinct subtypes in two- and three-class tasks, further optimization is needed for multi-class classification. After testing random repeats and folds across the SBC dataset, the model with the best learning rate and weight decay combination on average was found and is shown in Table 3.

The confusion matrices show the results from individual test sets of a specific fold, as well as a specific repeat within our CV process (Figure 2). The results suggest that the model performs well in classifying certain molecular subtypes, particularly luminal A and “Other” cases, with high accuracy. However, it struggles more with distinguishing between HER2(+), luminal B, and TNBC, leading to some misclassification errors.

**Figure 3:**
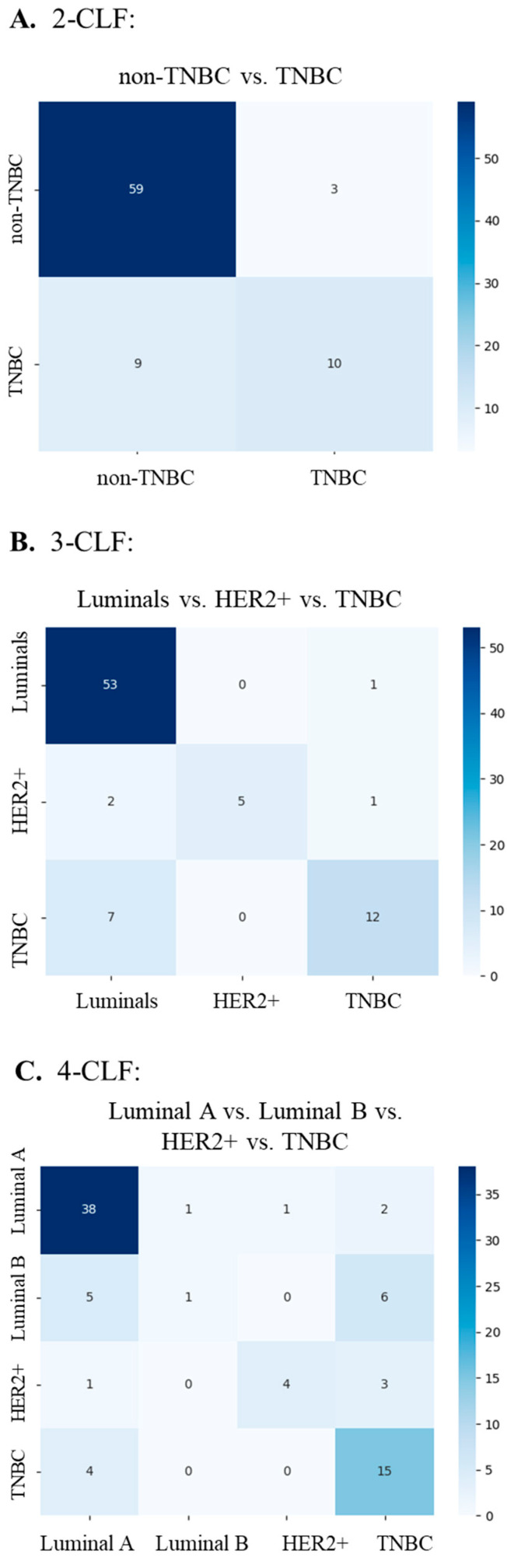
The confusion matrices on the validation set of the SBC dataset for (**A**) 2-CLF (non-TNBC vs. TNBC), (**B**) 3-CLF (Luminals vs. HER2+ vs. TNBC), and (**C**) 4-CLF (Luminal A vs. Luminal B vs. HER2+ vs. TNBC).

Figure 3A reveals that the 2-CLF model performed well in classifying non-TNBC with minimal errors, with 59 correct predictions and only 3 false positives. However, TNBC classification is less reliable, with nine false negatives. This suggests an imbalance in classification, where the model favors predicting non-TNBC over TNBC. Figure 3B shows that the 3-CLF model performed well in classifying the luminal subtype, correctly identifying 53 out of 62 samples, resulting in high specificity. However, the model struggled more in distinguishing HER2(+) and TNBC cases. Figure 3C demonstrates that the 4-CLF model achieved strong performance in classifying luminal A, correctly predicting 38 samples, but it struggled more with luminal B, HER2(+), and TNBC classifications. There were several misclassifications between these subtypes, indicating that their features may overlap, making differentiation more difficult.

## 4. Discussion

BC molecular subtypes vary in behavior, treatment response, and prognosis, making accurate and reproducible evaluation essential in clinical practice. Most pathology laboratories rely on IHC surrogate classification for BC molecular subtyping. However, this method faces challenges such as a lack of standardized IHC procedures and cut-off values, observer-related variability, and a lack of reproducibility. On the other hand, IHC surrogates do not always accurately reflect the true intrinsic molecular subtypes and show poor-to-moderate concordance with PAM50, with misclassification rates reaching up to 30–40% across all subtypes [17,18,19]. Gene expression has its advantages but also comes with constraints, such as higher costs and longer processing times. Considering the promising results of deep learning in cancer research, particularly in classification and prediction, it is important to evaluate it as a third approach. Moreover, AI-driven methods can overcome the limitations of the other two approaches mentioned above. In this study, we validated an AI method for determining BC molecular subtypes directly from H&E-stained WSIs. The method used the pretrained BCNB public dataset and was externally validated using the SBC dataset, which shows its robustness against different image acquisition variations.

The results show that the best outcome is achieved with the 2-CLF task, which distinguishes between TNBC and non-TNBC. This is an important step for the next phase of the study, which aims to predict prognosis specifically within the TNBC subgroup. In multiclass classification, performance can be improved by enhancing recall, which can be achieved by adjusting class weights or using more balanced data. The difficulty in distinguishing between luminal A and luminal B cases largely depends on proliferation and therefore, integrating an automatic proliferation assessment based on mitosis localization could help accurately differentiate between these two subtypes [20]. The AI model used in our study focuses solely on tumor cells and does not account for the relationship with the tumor microenvironment (TME). While this could be considered a limitation, it should be noted that the surrogate model, namely molecular subtyping with IHC, also only evaluates tumor cells.

This study focuses on BC using H&E WSIs, which helps avoid the challenges associated with non-standardized IHC procedures and observer variability. Additionally, H&E-based approaches are generally more cost-effective compared to IHC and provide results in a shorter time. In particular, in low-income countries, the IHC method is not affordable for hospitals, and patients are often treated without molecular subtyping. In this case, the method we propose might be highly beneficial, as even a small, single-slide scanner could surpass the need for the IHC method, which requires significant capital investment. Moreover, with the emergence of new techniques that allow for the virtual staining of unstained (non-H&E-stained) slides, interlaboratory variability can also be resolved more quickly and cost-effectively [21]. Different approaches have been studied to investigate molecular subtype prediction in BC, especially using image analysis or deep learning frameworks, particularly with H&E-stained images, presenting reliable and promising results [22,23,24,25]. However, research involving validation with real-world data is limited, and it is crucial for improving the generalizability and reproducibility of these methods for clinical use.

Considering the increasing importance of neoadjuvant therapies, BC diagnosis is increasingly performed using core-needle biopsies, which may not fully capture the tumor heterogeneity observed in surgical resections. Therefore, the use of the SBC dataset for validation was highly significant. Since there is no specific clinically validated morphological feature that AI can learn from BC H&E to distinguish molecular subtypes, utilizing MIL, which relies solely on slide-level labeling rather than patch- or pixel-level annotations, is crucial for enhancing the model’s ability to capture relevant patterns and improve classification performance. One of the discussed molecular subtypes, TNBC, is known to have multiple molecular subtypes, each with distinct prognoses [26,27]. Despite these differences, all subtypes currently receive the same treatment, highlighting the importance of molecular subtyping. One such subtype is the luminal androgen receptor (LAR) subtype, characterized by AR-positive TNBC cases, for which studies have shown the effectiveness of anti-androgen therapy [28]. Another subtype is characterized by a high abundance of immune cells, and studies have demonstrated that tumor-infiltrating lymphocytes (TILs) are prognostic in TNBC [29]. This group is particularly valuable due to the potential for immunotherapy in its treatment. As a plan, we propose evaluating our model for TNBC subtyping to further explore its potential.

There are, however, some limitations to the current study. There was a clear class imbalance within the dataset. This study used a population-based dataset, reflecting BC prevalence in the population, which is one of the key aspects that distinguishes our study from others; however, this also means that class imbalance is inevitable. To address this imbalance, optimizing the model weights using a weighted categorical cross-entropy loss function is essential, as was done in our study. Moreover, the current study was analyzed using the surrogate method, but the most reliable and accurate findings should be obtained by comparing gene expression analyses and this proposed AI method. Numerous commercial gene panels are available, including Oncotype DX, MammaPrint, Prosigna (PAM50), and EndoPredict. PAM50 is currently widely used in Norway as a transcriptome-based classification system for intrinsic subtype classification [30]; it was implemented during the EMIT study. As of December 2024, recruitment for this study is now closed, and more than two thousand BC patients analyzed using the PAM50 test and the surrogate IHC classification system. Oncologists based their treatment decision first on IHC and then on PAM50 [31]. Another limitation of this study is the exclusion of HER2 2+ cases. Using studies where all cases are subtyped through gene analysis, regardless of HER2 status, could help address this limitation [31]. The prevalence of TNBC in the population is around 15%, but in our dataset, it was slightly higher than expected. Excluding HER2 2+ cases or obtaining false negative receptor results in older tissue blocks may explain this. Older blocks can lead to misclassifying non-TNBC cases as TNBC due to antigen degradation or suboptimal staining. HER2-low tumors are characterized by low HER2 protein expression [IHC 1+ or IHC 2+ with negative in situ hybridization (ISH)] but without HER2 gene amplification, highlighting the complexity of BC. These tumors were previously considered part of the HER2-negative group and have gained attention due to their potential responsiveness to novel HER2-targeted therapies. Its prognosis is being actively investigated, and it is believed that different treatment strategies may be necessary for this subgroup. Validating the proposed model on datasets with available PAM50 results is important to better address this uncertain and evolving area. Furthermore, the use of different digital scanners in this dataset has helped address variabilities related to image acquisition. Although all slides were stain-normalized to a consistent color distribution, one of the limitations of the study is that model performance was not evaluated across the different scanner types.

This study is valuable, as the model provides slide-level predictions of molecular subtyping using only H&E-stained WSIs and represents, to our knowledge, the first validation of such a model using a large, population-based, real-world BC dataset. Only relying on H&E WSIs offers an easily applicable alternative and has the potential to shorten time to diagnosis.

## 5. Conclusions

In conclusion, our study demonstrated that the proposed AI model helped to distinguish BC molecular subtypes on H&E slides but only with distinct subtypes. The results are promising; however, future improvements may require improving performance in multiclass classification. Using only H&E-stained slides is undoubtedly important for pathology laboratories with a high workload. Future studies should focus on addressing the limitations described above and ensuring the approach is robust and applicable in clinical settings for personalized treatment.

## Figures and Tables

**Table 1:** Surrogate classification of breast cancer based on IHC.

	ER	PR	HER2	Proliferation
**Luminal A**	positive	positive	negative	low
**Luminal B**	positive	any	any	high
**HER2-positive**	negative	negative	overexpressed	-
**TNBC**	negative	negative	negative	-

**Table 2:** Different molecular subtype classification tasks (CLF).

Classification Tasks	Description
Two-class task (2-CLF)	Non-TNBC TNBC
Three-class task (3-CLF)	Luminals (Luminal A and B) HER2-positive TNBC
Four-class task (4-CLF)	Luminal A Luminal B HER2-positive TNBC

**Table 3:** Average and standard deviation results of the best overall results for the three different classification tasks.

Task	AUC	Accuracy	F1 Score	Precision	Recall	Learning Rate	Weight Decay
2-CLF	0.823 ± 0.040	0.833 ± 0.015	0.824 ± 0.017	0.823 ± 0.018	0.833 ± 0.015	0.01	1 × 10^−6^
3-CLF	0.834 ± 0.037	0.795 ± 0.034	0.770 ± 0.046	0.761 ± 0.063	0.795 ± 0.034	0.01	0.01
4-CLF	0.790 ± 0.026	0.642 ± 0.043	0.601 ± 0.045	0.587 ± 0.056	0.642 ± 0.043	0.01	1 × 10^−6^

## Data Availability

The dataset generated during the current study is not publicly available because of ethical and legal concerns. Anonymized data can be made available from the Stavanger University Hospital Institutional Data Access/Ethics Committee (contact via email: rek-vest@uib.no, REK vest, Rogaland, Vestland, Norway) for researchers who meet the criteria for access to confidential data.

## Abbreviations

The following abbreviations are used in this manuscript:

AI	Artificial Intelligence
AUC	Area Under the Curve
BC	Breast Cancer
BCNB	Breast Cancer Needle Biopsy
CLF	Classification tasks
CNN	Convolutional Neural Network
CV	Cross-Validation
ER	Estrogen Receptor
H&E	Hematoxylin and Eosin
HER2	Human Epidermal Growth Factor Receptor 2
IHC	Immunohistochemistry
ISH	In Situ Hybridization
LAR	Luminal Androgen Receptor
MAI	Mitotic Activity Index
MIL	Multiple Instance Learning
NBCG	Norwegian Breast cancer Group
PAM50	Prediction Analysis of Microarray 50
PR	Progesterone Receptor
SBC	Stavanger Breast Cancer
TILs	Tumor-Infiltrating Lymphocytes
TME	Tumor Microenvironment
TNBC	Triple-Negative Breast Cancer
WSI	Whole Slide Image
